# Radiographic Evidence of Nonoccupational Asbestos Exposure from Processing Libby Vermiculite in Minneapolis, Minnesota

**DOI:** 10.1289/ehp.1103529

**Published:** 2011-10-12

**Authors:** Bruce H. Alexander, Katherine K. Raleigh, Jean Johnson, Jeffrey H. Mandel, John L. Adgate, Gurumurthy Ramachandran, Rita B. Messing, Tannie Eshenaur, Allan Williams

**Affiliations:** 1 Division of Environmental Health Sciences, School of Public Health, University of Minnesota, Minneapolis, Minnesota, USA; 2 Minnesota Department of Health, St. Paul, Minnesota, USA; 3 Department of Environmental and Occupational Health, Colorado School of Public Health, Aurora, Colorado, USA

**Keywords:** amphibole, asbestos, community exposure, Libby vermiculite, lung diseases, pleural abnormalities

## Abstract

Background: Community exposure to asbestos from contaminated vermiculite ore from Libby, Montana, occurred in many processing sites in the United States, including a densely populated urban residential neighborhood of Minneapolis, Minnesota.

Objective: We examined exposed community residents who never worked at the plant or never lived with a plant worker for radiographic evidence of lung changes consistent with asbestos exposure.

Methods: We obtained posteroanterior chest radiographs to identify the prevalence of pleural abnormalities consistent with pneumoconiosis, as determined by consensus of two National Institute for Occupational Safety and Health–certified B-reader radiologists. We estimated cumulative asbestos exposure (fibers per cubic centimeters × months) with air dispersion model data and activity-based modeled exposure estimates for vermiculite processing waste contact. We modeled associations between pleural abnormalities and asbestos exposure using multiple logistic regression to adjust for year of birth, sex, and potential occupational asbestos exposure.

Results: Radiographs were obtained for 461 participants. The prevalence of pleural abnormalities by B-reader consensus was 10.8%. A history of direct contact with the waste and ever playing in the waste piles was associated with pleural abnormalities {odds ratio [OR] 2.78 [95% confidence interval (CI): 1.26, 6.10] and 2.17 (95% CI: 0.99, 4.78), respectively, when adjusted for background exposure}. The regression coefficients for log-transformed measures (fibers per cubic centimeters × months) of background exposure and activity-based exposure were 0.322 (95% CI: 0.078, 0.567) and 0.063 (95% CI: –0.013, 0.139), respectively, when adjusted for each other, and 0.283 (95% CI: 0.104, 0.463) for cumulative exposure from all sources.

Conclusion: These results support the hypothesis that community exposure to asbestos-contaminated vermiculite originating from Libby, Montana, is associated with measurable effects based on radiographic evidence.

Vermiculite ore from a mine near Libby, Montana, is known to be contaminated with amphibole asbestos (primarily winchite, richterite, and tremolite), which is liberated in the mining and processing of the ore [Agency for Toxic Substances and Disease Registry (ATSDR) 2008; Meeker et al. 2003]. The mining and processing of vermiculite in Libby have been associated with elevated mortality from lung cancer, nonmalignant respiratory disease, and mesothelioma (Amandus and Wheeler 1987; McDonald et al. 1986a; Sullivan 2007) in exposed workers, members of the community, and workers employed at distant vermiculite processing sites (Amandus et al. 1987; McDonald et al. 1986b; Peipins et al. 2003; Rohs et al. 2008). Environmental contamination from this source is not limited to Libby, as hundreds of thousands of tons of the ore were shipped throughout the country for processing. One of the destinations for the Libby vermiculite ore was the Western Minerals/W.R. Grace (WM/WRG) facility in Minneapolis, Minnesota.

The WM/WRG facility processed vermiculite ore mined in Libby, Montana, from 1938 to 1989. This facility was one of approximately 250 such processing facilities in the United States that received Libby ore. At the WM/WRG facility, the vermiculite ore was heated in two furnaces and expanded in a process known as exfoliation to make Zonolite® insulation, Monokote® fireproofing, and other building materials. In addition to the vermiculite product, the exfoliation produced a waste material that contained up to 10% amphibole asbestos [Minnesota Department of Health (MDH) 2005]. The waste rock was piled on the WM/WRG property and offered freely to the community. A unique aspect of the WM/WRG exfoliation site in Minneapolis is its location in a residential neighborhood: a mix of single-family homes, multifamily homes, schools, and churches. Neighborhood residents hauled the rock from the piles and used it for gardening and as fill material for driveways and yards. Neighborhood children also played on the piles of vermiculite processing waste, as access to the site was not restricted. The proximity of the neighborhood to the plant and the opportunities for contact with the waste rock created a scenario for substantial nonoccupational exposure to asbestos.

Responding to residential concern in 2000 (Gordon 2000), the MDH and ATSDR initiated the Northeast Minneapolis Community Vermiculite Investigation (NMCVI). The NMCVI staff surveyed > 1,600 properties and worked with the U.S. Environmental Protection Agency (EPA) to document contamination on > 260 properties, which subsequently were cleaned by the U.S. EPA in a time-critical removal under the federal Superfund program. The MDH/ATSDR investigation assessed past and present Libby asbestos exposure pathways for workers and community members, including relative fiber levels and exposure duration (MDH 2005)in addition to estimating ambient air exposures during the years the plant was operating (Kelly et al. 2006; MDH 2003). Interviews were conducted with > 6,400 community members to establish baseline information. As a follow-up to the exposure characterization in this community, we evaluated the impact of nonoccupational exposure to asbestos from contaminated vermiculite on lung health as evidenced by radiographic changes in the lungs of community members.

## Materials and Methods

Approval for the study protocol was obtained from the human subjects committees of the University of Minnesota and the MDH. All participants were guided through an informed consent process, and written consent was obtained before their participation.

*Study population.* The focus of this study was nonoccupational exposure to asbestos from contaminated vermiculite in the population surrounding the WM/WRG site in Minneapolis. We identified 2,222 members of the original NMCVI cohort who were never employed at the WM/WRG plant, were not a household contact of WM/WRG employees, and were first at risk of exposure to the contaminated vermiculite before 1980. The latter inclusion criterion ensured an adequate latency period for effects of the asbestos exposure to become apparent.

To obtain a sample representative of the range of community exposures, the population was stratified into groups to represent three exposure scenarios: *a*) intense intermittent exposure, *b*) long-term high ambient background exposure, and *c*) low ambient background exposure. We classified people with a childhood history of playing in the piles of waste rock outside the plant as the group with intense intermittent exposures to potentially high concentrations of asbestos fibers. The long-term high and low background exposure groups were selected based on residential history and frequency matched to the age distribution of the intense intermittent exposure category. All members of the cohort were classified by cumulative exposure experienced before 1 January 1980. The exposure estimates were based on the period-specific air dispersion model developed by the Minnesota Pollution Control Agency and the MDH and on residential history. The initial model estimated exposure based on the amount of time the individual lived in the affected area multiplied by the yearly average plant emissions rates (Kelly et al. 2006; MDH 2003). Excluding persons identified as having intense intermittent exposure and those who did not have appropriate residence data, a total of 1,839 individuals were classifiable by background exposure. The long-term high background exposure group was initially identified as the upper quartile of this exposure distribution. The low-exposure category was selected from the lowest quartile of this distribution. When the available samples from the upper and lower quartile were exhausted, the second and third quartiles were entered into the selection protocol. This selection protocol ensured a distribution of estimated exposure.

The institutional review board protocol required MDH, as the institution of record for the NMCVI, to initiate contact. MDH forwarded the names and contact information of individuals interested in learning more about the study to the University of Minnesota. The potential participants were sent a letter and study brochure, along with consent and HIPAA (Health Insurance Portability and Accountability Act) forms. This was followed with a call from a trained telephone interviewer to review the study purpose, procedures, requirements, consent, and HIPAA forms and to answer any questions. Those who chose to participate signed and returned the consent and HIPAA forms to the study office.

Upon receiving the signed consent and HIPAA forms, a second packet containing a cover letter, clinic information, and questionnaire was mailed to each participant. An interviewer followed-up by telephone to ensure the instructions and procedures were understood and to encourage the participant to promptly attend a participating clinic and return the completed questionnaire.

*Radiographic measurements.* Participants attended a local contract medical clinic that specializes in occupational health screening and could perform plain-film X-rays for B-reading. The clinics offered evening and weekend appointments to accommodate various schedules. The prevalence of pleural abnormalities, parenchymal opacities, and other evidence of asbestosis was determined with a single posteroanterior (PA) radiograph following the recommendations of the International Labour Organization (ILO 2002).

The radiographs were reviewed and scored by National Institute for Occupational Safety and Health–certified B-readers using standard methodology (ILO 2002). The B-readers were board-certified, practicing radiologists from a practice in the Minneapolis–St. Paul metropolitan area. The presence of pleural abnormalities consistent with pneumoconiosis, both pleural thickening and pleural plaques, was the primary indicator of asbestos-induced changes in the lungs. The final classification of radiographic changes consistent with pneumoconiosis required consensus by two B-readers. If there were discrepancies between two initial readers regarding pleural abnormalities or the presence of parenchymal abnormalities with an ILO classification of 1/0 or above, the radiographs were reviewed by a third certified B-reader. The clinical interpretations were summarized and sent to the participants and their health care provider if requested.

*Exposure assessment.* We assessed exposure to asbestos fibers through ambient exposure and activities that brought participants in contact with the waste rock. The details of the exposure assessment are summarized here and reported in full elsewhere (Adgate et al. 2011). Briefly, background exposure was determined by length of residence in the affected community and estimates of airborne fiber concentration. The MDH study ascertained residential histories within the affected community for all study participants (Kelly et al. 2006). Air dispersion modeling and deposition were used to characterize cumulative potential asbestos background exposure for this population (Kelly et al. 2006; MDH 2005). The models evaluating facility emissions and resulting air concentrations and deposition accounted for prevailing weather patterns, emission controls installed in the plant, and post-shutdown emissions due to fugitive dust and construction activities. Ambient fiber concentrations during peak production years before installation of air pollution control equipment (1938–1972) varied by several orders of magnitude, with concentrations ranging from 0.026 fibers/cm^3^ (f/cc) within one to two blocks of the plant to 0.0001 f/cc at the margins of the neighborhood. The exposures for this study were estimated for the period of plant operation (1938–1989). Cumulative background exposure (CBG; fibers per cubic centimeter × months) was estimated by summing duration and period-specific airborne fiber concentration for each subject:

CBG*_i_* = ∑BG*_ij_T_j_* , [1]

where BG*_ij_* is monthly average airborne asbestos fiber concentration (fibers per cubic centimeter) for subject *i* from the air dispersion model during the *j*th time period, and *T_j_* is the number of months subject *i* lived in the impact zone of WM/WRG plant emissions.

Potential activity-based exposure pathways were ascertained by questionnaire for the NMCVI study. The interviews solicited self-reports of direct contact from moving waste rock from WM/WRG plant, using waste rock at home (e.g., on their lawns or gardens), installing or removing vermiculite insulation, and playing in or around waste piles at the plant. Exposures were estimated based on probable ranges of the activity durations, the frequency of the activities, and ranges of potential airborne fiber concentrations. The potential airborne fiber concentrations were derived from experimental data that reconstructed these activities in the U.S. EPA Libby investigation (Weis 2001a, 2001b). Uniform distributions were assumed based on the following ranges for each activity: moving waste rock from plant, 0.07–0.14 f/cc; using waste rock at home, 0.02–0.227 f/cc; installing or removing vermiculite insulation, 0.142–0.568 f/cc; and playing in or around waste piles at the plant, 0.14–1.72 f/cc. Monte Carlo simulation software (Crystal Ball 2000, version 2.2; Decisioneering, Boulder, CO) was used to estimate the probability distribution of asbestos exposures (fibers per cubic centimeter × months) for each activity (*k*) based on the assumption that during these activities each individual was exposed to a range of asbestos concentrations with varying exposure durations that ranged from 0.25 to 8 hr. The cumulative activity concentration (CAC; fibers per cubic centimeter × months) for each activity (*k*) was estimated as follows:

CAC*_ik_* = (*AC* × *T* × *F* × 12) ÷ (24 × 365), [2]

where for each individual *i*, *AC* is activity-specific asbestos concentration in air (fibers per cubic centimeter), *T* is exposure duration (hours/day), and *F* is frequency the activity occurred (total days). The expression *AC_k_* × *T_k_* × *F_k_* has units of fibers per cubic centimeter × hours. The remainder of the expression [12 months/year ÷ (24 hr/day × 365 days/year)] converts the result to fibers per cubic centimeter × months.

The total cumulative asbestos exposure (TCE; fibers per cubic centimeter × months) for each individual is the sum of the estimated background levels and the median values of the simulation output distribution for each individual:

TCE*_i_* = ∑*_k_*CBG*_i_* + CAC*_ik_* . [3]

To focus on the community exposure, the population for this study explicitly excluded employees of WM/WRG and their household members. However, other occupations also have the potential for asbestos exposure. To characterize this potential exposure, we used responses from the NMCVI questionnaire that asked about ever employment in 15 occupations that have potential for asbestos exposure.

*Statistical analysis.* The prevalence odds ratios (ORs) of pleural changes were modeled with multivariate logistic regression for the following exposure metrics: background exposure, total activity-based exposure, exposure acquired as a child when playing on the waste piles, and total exposure. We used cumulative exposure metrics, expressed as fibers per cubic centimeter × months. The exposure data were log-normally distributed, and model fit, as determined by likelihood ratio statistics, was improved by using the log-transformed variable. We categorized the exposure data as < 50th, 50th–75th, and > 75th percentiles for background, total exposure, and activity exposure for those with any activity, respectively. The models were adjusted for sex, year of birth, and ever holding a job with potential asbestos exposure as reported on the NMCVI questionnaire. Missing exposure data were imputed with a multiple imputation algorithm (SAS MI procedure, version 9.2; SAS Institute Inc., Cary, NC) based on the exposure data for all eligible persons (*n* = 2,222), creating 18 replicate imputed data sets. We imputed missing data for activity-based exposures for only those individuals reporting the activity. For descriptive purposes, we summarized the imputed data with the median value for each parameter. Logistic regression models using the 18 imputed data sets were summarized using the MIANALYZE procedure in SAS (version 9.2). Prevalence ORs are reported to describe the associations, and 95% Wald’s confidence intervals (CIs) are presented to characterize the precision of the estimates.

## Results

From the original 2,222 persons eligible, 1,765 were randomly selected from the exposure groups and were eligible for contact by the MDH, and 1,133 assented to be contacted by the University of Minnesota. Of the remaining 632, 68 were deceased, 367 declined, and 197 could not be reached. A total of 698 consented to participate, and 461 completed the clinic visit. Compared with nonparticipants, those who attended the clinic were more likely to be male, born after 1940, and nonsmokers and to have activities that would lead to exposure (Table 1). The percentage of participants and nonparticipants who reported having an asbestos-exposed job was essentially the same. Playing on the waste piles was reported by 180 (39%) of the 461 participants, and 223 (48%) reported at least one activity resulting in exposure to the waste material.

**Table 1:** Characteristics of the original eligible study population (2,222 persons) who did and did not attend the clinic [*n* (%)].

Attended clinic		
Characteristic	Yes	No
Total		461		1,761
Sex				
Male		241 (52.3)		860 (48.8)
Female		220 (47.7)		901 (51.2)
Birth year				
≤ 1940		89 (19.3)		654 (37.1)
1941–1950		139 (30.2)		374 (21.2)
1951–1960		148 (32.1)		385 (21.9)
≥ 1960		85 (18.4)		348 (19.8)
Ever a regular smoker		242 (52.5)		1,040 (59.1)
Played on waste piles at plant		180 (39.0)		401 (22.8)
Moved waste from WM/WRG plant		51 (11.1)		126 (7.2)
Used waste at home, lawn, or garden		44 (9.5)		109 (6.2)
Installed/removed vermiculite insulation		44 (9.5)		129 (7.3)
Any activities leading to exposure		223 (48.4)		569 (32.3)
Ever held any asbestos-exposed job		129 (28.0)		479 (27.2)

Prevalence of pleural abnormalities consistent with pneumoconiosis reported by any B-reader was 15.4% (Table 2). A consensus determination of pleural abnormalities was made for 10.6% of the participants. Seven participants had X-rays of insufficient quality for B-reads; thus, the final prevalence of consensus abnormalities was 10.8%. Consensus readings of diffuse pleural thickening and pleural plaques were reported in 5 and 45 participants, respectively. Parenchymal abnormalities equal to or exceeding ILO category 1/0 were recorded by at least one B-reader in 18 participants, and 9 of these had the consensus of two readers. One of these had a film coded as unreadable by one B-reader, so 8 were clearly ILO ≥ 1/0. There were too few parenchymal abnormalities to statistically evaluate, but of the 8 with a consensus of ≥ 1/0, all had some evidence of exposure through an activity, 6 of which reported playing in the waste piles. The 8 individuals were distributed across the background and total exposure estimates, with 3, 2, and 3 in each of the upper three quartiles. Half of the parenchymal abnormalities also reported having a job with potential asbestos exposure.

**Table 2:** Summary of radiographic results for all participants attending the clinic (*n* = 461).

Radiograph interpretation	*n* (%)
Any pleural abnormality*a*	71 (15.4)
Pleural abnormality,*a* consensus of two readers	49 (10.6)
Diffuse pleural thickening, consensus of two readers	5 (1.1)
Any pleural plaques observed	70 (15.2)
Pleural plaques, consensus of two readers	45 (9.8)
Any parenchymal abnormality*b*	18 (3.9)
Parenchymal abnormality,*b* consensus of two readers	9 (2.0)
**a**Consistent with pneumoconiosis. **b**At ILO ≥ 1/0.	

Participants diagnosed with pleural thickening or pleural plaques were older and more likely to be male, to have held an asbestos-exposed job, and to report activities associated with contaminated vermiculite contact (Table 3). The exposure distribution for background exposure and activity-based exposure followed a pattern of higher exposure in those with pleural abnormalities.

**Table 3:** Characteristics of study participants with and without pleural abnormalities.

Pleural abnormalities*a*		
Characteristic	Yes	No
Total*b*		49 (100)		405 (100)
Sex				
Male		39 (79.6)		198 (48.9)
Female		10 (20.4)		207 (51.1)
Birth year				
≤ 1940		19 (38.8)		67 (16.5)
1941–1950		20 (40.8)		116 (28.6)
1951–1960		8 (16.3)		140 (34.6)
≥ 1960		2 (4.1)		82 (20.2)
Played on waste piles		28 (57.1)		147 (36.3)
Moved waste from WM/WRG plant		13 (26.5)		37 (9.1)
Used waste at home, lawn, or garden		8 (16.3)		34 (8.4)
Installed/removed vermiculite insulation		8 (16.3)		35 (8.6)
Any contact with the waste		35 (71.4)		182 (44.9)
Any activities leading to exposure		33 (67.3)		167 (41.2)
Any asbestos-exposed job		22 (44.9)		105 (25.9)
Total exposure, f/cc × months [median (IQR)]		0.202 (0.032–0.480)		0.049 (0.012–0.202)
Background exposure [median (IQR)]		0.079 (0.023–0.161)		0.028 (0.008–0.066)
Activity-based exposure [median (IQR)]		0.013 (0.0–2.72)		0.0 (0.0–0.079)
Values are *n* (%), except as noted. **a**Consensus by two B-readers. **b**Excludes 7 cases with X-rays that could not be classified by B-readers.				

In models adjusting for age, sex, and history of any asbestos-exposed job, the log-transformed measures of background exposure, exposure from pile playing, total activity exposure, and total exposure were positively associated with pleural abnormalities (Table 4). When both background and activity-based metrics were in the model, the background exposure maintained the strongest association with pleural abnormalities. The OR for a difference equivalent to the interquartile range (IQR) of total exposure (IQR = 0.014–0.245 = 0.231 f/cc-months) was 2.22 (95% CI: 1.34, 3.68).

**Table 4:** Regression coefficients from logistic regression models for associations between prevalence of pleural abnormalities and log concentration of background, activity, and total exposure.

Model	Parameter	β-Estimate*a* ± SE (95% CI)	*p*-Value
1		Background exposure		0.401 ± 0.120 (0.165, 0.637)		0.001
2		Total activity exposure		0.098 ± 0.036 (0.028, 0.168)		0.006
3		Cumulative exposure		0.283 ± 0.091 (0.104, 0.463)		0.002
4		Background exposure		0.322 ± 0.125 (0.078, 0.567)		0.009
		Total activity exposure		0.063 ± 0.039 (–0.013, 0.139)		0.102
5		Pile-playing exposure		0.080 ± 0.030 (0.021, 0.139)		0.008
6		Pile-playing exposure		0.042 ± 0.034 (–0.025, 0.109)		0.221
		Background exposure		0.331 ± 0.130 (0.076, 0.585)		0.011
**a**Adjusted for year of birth, history of having an asbestos-exposed job, and sex.						

When evaluated categorically, the association between pleural abnormalities and background exposure appeared limited to exposure at or above the 75th percentile (Table 5). Persons reporting ever having contact with the waste rock or ever playing on waste piles had ORs of 3.60 (95% CI: 1.71, 7.58) and 3.23 (95% CI: 1.59, 6.52), respectively. When background exposure was added as a covariate, ORs decreased to 2.78 (95% CI: 1.26, 6.10) and 2.17 (95% CI: 0.99, 4.78), respectively. When examined by exposure percentiles, the strength of association increased in the unadjusted models, but a monotonic exposure response beyond the 50th percentile was not evident in the adjusted models. The ORs for the third and fourth quartiles, compared with exposures below the median, were also attenuated when the background exposure was accounted for. When all sources of exposure were combined, the prevalence ORs were 3.79 (95% CI: 1.76, 8.16) and 1.05 (95% CI: 0.44, 2.50), respectively, for the fourth and third quartiles of exposure compared with exposures below the median.

**Table 5:** Number of pleural abnormalities (*n* = 49) and prevalence OR estimates by categorical exposure level for background exposure, activity-based exposure, and total exposure [OR (95% CI)].

Exposure	*n*	Unadjusted*a*	Adjusted*b*
Any contact with waste						
No (referent)		14		1		1
Yes		35		3.60 (1.71, 7.58)		2.78 (1.26, 6.10)
Ever played on piles						
No (referent)		21		1		1
Yes		28		3.23 (1.59, 6.52)		2.17 (0.99, 4.78)
Activity exposure (f/cc × months)*c*						
< 0.082		32		1		1
0.082 to < 0.422		9		2.04 (0.83, 5.00)		1.34 (0.52, 3.44)
≥ 0.422		8		2.36 (0.92, 6.05)		1.37 (0.49, 3.83)
Pile-playing exposure (f/cc × months)*c*						
< 0.158		36		1		1
0.158 to < 0.549		6		1.45 (0.53, 3.98)		0.97 (0.34, 2.77)
≥ 0.549		7		2.49 (1.75, 9.41)		1.31 (0.44, 3.85)
Background exposure (f/cc × months)*d*						
< 0.034		16		1		1
0.034 to < 0.077		8		0.91 (0.36, 2.31)		0.85 (0.32, 2.11)
≥ 0.077		25		3.18 (1.53, 6.59)		2.52 (1.15, 5.50)
Total exposure (f/cc × months)*d*						
< 0.0523		17		—		1
0.0523 to < 0.245		11		—		1.05 (0.44, 2.50)
≥ 0.245		21		—		3.79 (1.76, 8.16)
Sex						
Female		10		—		1
Male		39		—		3.8 (1.6, 8.9)
Exposed job						
No		22		—		1
Yes		27		—		1.31 (0.63, 2.73)
**a**Unadjusted for corresponding exposure covariates. All models are adjusted for year of birth, sex, and history of an asbestos-exposed job. **b**Adjusted for corresponding exposure. Activity-based exposure is adjusted for background exposure, and background exposure is adjusted for total activity exposure. **c**Cut points are based on the 50th and 75th percentile of those with any activity. All participants without activity are included in the referent category. **d**Cut points are at the 50th and 75th percentile of the exposure distribution.						

## Discussion

We observed an association between the prevalence of pleural abnormalities and estimates of environmental exposure to asbestos fibers attributed to processing Libby vermiculite. These results support the hypothesis that nonoccupational community exposure to asbestos fibers from the processing of Libby vermiculite produced measurable effects. Associations were most consistent with long-term, lower exposure, as represented by background exposure, in contrast to more intense but intermittent exposure from specific activities.

These results must be considered in context with the following limitations of the study. The study is small, which limited estimate precision and our ability to explore more complex exposure models. The requirement of attending a clinic for an X-ray, with no explicit benefit to the participant, likely limited study participation, and it is possible that participation was influenced by known health status. However, arguments may be constructed to favor a positive or negative bias in the results. Our metrics of activity-based exposure were derived from self-reported activities as assessed in 2001 when the cohort was enumerated. Although this provided excellent baseline information for the whole cohort, assumptions about the duration of the activity were needed, which likely introduced some exposure misclassification. An air dispersion model was used for retrospective estimation of background exposure based on residential history and proximity to the vermiculite plant. This model assumes an equivalent exposure opportunity for all people, regardless of where they spent their day. Assessment of radiographic pleural abnormalities was accomplished with a single PA chest radiograph, and although this is a standard screening practice, pleural abnormalities may be missed without evaluating multiple views. More sensitive assessments of pleural abnormalities can be done with computed tomography (CT) scans; however, cost, potentially unnecessary exposure to ionizing radiation, and risk of frequent false-positive findings reduce the utility of CT scans for screening low-risk individuals. False-positive misclassification was reduced by accepting pleural abnormalities only when there was consensus by two B-readers. The initial assessment of the two primary readers detected 71 pleural abnormalities consistent with pneumoconiosis, whereas the consensus readings only found 49. In a number of the disputed assessments of pleural abnormalities ultimately classified as normal, the clinic notes from the dissenting B-reader postulated the presence of pleural shadows from fat. Associations were attenuated if the 22 participants with disputed readings were classified as having pleural abnormalities. For example, the regression coefficient for background exposure was reduced from 0.401 (95% CI: 0.165, 0.637) to 0.146 (95% CI: –0.091, 0.382), and cumulative exposure was reduced from 0.283 (CI: 0.104, 0.463) to 0.168 (95% CI: 0.014, 0.323).

An estimated 138,000 tons of Libby ore were processed at the WM/WRG plant in Minneapolis. The resulting emissions and subsequent use of the waste rock contaminated areas up to 2 miles from the plant (Kelly et al. 2006). Our exposure models combined ambient dispersion and activity-based estimates that demonstrate substantial nonoccupational asbestos exposures for residents of this neighborhood, albeit with considerable variability by exposure pathway (Adgate et al. 2011). Although median exposure estimates for the cohort were driven by direct plant emissions, contact with waste rock resulted in substantial additional exposures to some individuals. Most important, playing on piles of waste rock was the largest contributor to high-end exposures for the subpopulation of individuals who engaged in these activities.

Previous research in Libby has shown that playing on piles of waste rock is associated with changes in lung function and is a risk factor for asbestos-related disease (Horton et al. 2006, 2008; Peipins et al. 2003). Although these studies of retrospective community exposure to asbestos explored the relationship between Libby asbestos and various health effects, most of them did not provide individual estimates of cumulative exposure.

Our estimates of cumulative exposure concentrations (fibers per cubic centimeter × months) do not directly translate to the existing health-based standards. The current Occupational Safety and Health Administration (OSHA) permissible exposure limit, which has been reduced considerably in the last 40 years (ATSDR 2001), is 0.1 f/cc based on an 8-hr time-weighted average (OSHA 1995). The estimated median asbestos concentrations in U.S. cities range from 10^–8^ to 10^–4^ f/cc (ATSDR 2001). At 10^–4^ f/cc the range of cumulative exposure from 20 to 40 years would be 0.024–0.048 f/cc-month, which is in the lower end of the exposure distribution we describe. The median concentration for the participants with pleural abnormalities was an order of magnitude greater. Thus, exposure estimates developed for this study were well above urban background. When converted to a scale of fibers per cubic centimeters × year, the upper end of the exposure distribution for this study was within the lower quartile exposure ranges in which pleural abnormalities were observed in workers in Libby (Horton et al. 2008; Rohs et al. 2008).

The impact of Libby asbestos exposure on the health of employees of the Libby vermiculite mines and mills has been well documented. Mortality studies of W.R. Grace mine workers in Libby have found significantly increased mortality from nonmalignant respiratory disease and lung cancer (Amandus and Wheeler 1987). A more recent update of an occupational cohort of 406 vermiculite miners employed before 1963 and followed through 1999 reported an excess of lung cancer deaths [44 deaths; standardized mortality ratio (SMR) = 2.40], nonmalignant respiratory deaths (51 deaths; SMR = 3.09), and all-cause deaths (285 deaths; SMR = 1.27), which increased with cumulative exposure (McDonald et al. 2004). A total of 12 mesothelioma deaths had occurred by the end of 1999, representing 4.2% of 285 deaths in the cohort since July 1983. These associations continue to be corroborated through additional follow-up of this working population (Sullivan 2007). Health effects from occupational exposures to Libby asbestos in operations beyond Libby are emerging. Workers at a vermiculite expansion plant that produced material primarily for lawn care products were found to have increased risk of pleural abnormalities at exposures lower than those seen in Libby (Rohs et al. 2008).

Beginning in 2000, ATSDR began investigating the effects of occupational and nonoccupational exposure to Libby asbestos in the Libby community. A study of death certificates from 1979 through 1998 found a 40- to 60-fold elevation in asbestosis deaths in Libby (Horton et al. 2006). These deaths occurred primarily among former employees. A respiratory health screening investigation conducted in Libby, Montana (Peipins et al. 2003), found radiographic pleural and interstitial abnormalities associated with occupational exposure among former W.R. Grace employees. Among 365 employees screened, 51% had pleural abnormalities and 4% had interstitial abnormalities as determined by at least two B-readers. Among community members in Libby, the rates of pleural abnormalities were higher among males and increased with age, smoking history, and body mass. Increased prevalence of pleural abnormalities was also found among female household contacts of W.R. Grace employees (OR = 3.62; 95% CI: 2.70, 4.83) compared with females who were not household contacts. Participants living > 34 years in the Libby area also were at increased risk (OR = 2.12; 95% CI: 1.66, 2.70) compared with persons living < 14 years in the area. People who played on vermiculite waste piles frequently had twice the prevalence of pleural abnormalities as those who never played in the piles (OR = 2.02; 95% CI: 1.59, 2.57). Persons exposed through multiple exposure pathways were also at increased risk. Further evidence (Whitehouse et al. 2008) of potential nonoccupational health effects in Libby has recently been documented and includes 31 cases of mesothelioma that had no documented occupational exposure to asbestos but were exposed to emissions from the plant in Libby. A report (Whitehouse 2004) of 123 Libby patients with occupational and nonoccupational exposure indicated progressive loss of pulmonary function associated with pleural changes in 94 individuals.

Environmental and secondary occupational exposures to asbestos have been associated with malignant mesothelioma and benign pleural disease in several settings (Ferrante et al. 2007; Reid et al. 2008, 2009). Although these results support the notion that nonoccupational asbestos exposure has hazardous consequences, the exposures encountered were much higher than seen in our study.

Low-level persistent exposure to asbestos in other populations also has been associated with measurable changes in the lungs. “Endemic pleural plaques” have been described in areas of the world where mineral fibers occur naturally in soil or are used locally for whitewashing (Ates et al. 2010; Karakoca et al. 1998; Metintas et al. 2005; Yazicioglu 1976; Yazicioglu et al. 1980).

We report a stronger association between the prevalence of pleural changes and estimated cumulative exposure acquired over many years compared with higher intermittent peak exposures. The reason for this is not clear. It may speak to the scale of exposure estimates from activity-based exposure or an overestimate of the frequency of exposure through activities, such as pile playing. Alternatively, the fiber particles from persistent long-term, lower-level exposure from fugitive plant emissions, although correlated with the activity-based exposure, may be sufficiently different from the exposures in the waste rock that a greater proportion of the dust is deposited in the lower reaches of the respiratory tract. Our study, however, was too small to discern these differences.

In summary, we observed a consistent association between environmental asbestos exposure from the processing of Libby vermiculite and prevalent pleural abnormalities. These results suggest the effects of asbestos exposure may occur at lower levels than previously believed. Future research should concentrate on the effects of long-term, low-level exposure and adverse outcomes in nonoccupational, asbestos-exposed populations.

## Correction

In the “Results” section of the manuscript originally published online, OR (95% CI) values were transposed in two places: *a*) for persons reporting ever having contact with the waste rock or ever playing on the waste piles, and *b*) for the same categories when background exposure was added as a covariate. Data were presented correctly in Table 5. The text has been corrected here.
